# Carotid intima media thickness and blood biomarkers of atherosclerosis in patients after stroke or myocardial infarction

**DOI:** 10.3325/cmj.2016.57.548

**Published:** 2016-12

**Authors:** Iwona Kurkowska-Jastrzębska, Michał A. Karliński, Beata Błażejewska-Hyżorek, Iwona Sarzyńska-Długosz, Krzysztof J. Filipiak, Anna Członkowska

**Affiliations:** 1 2nd Department of Neurology, Institute of Psychiatry and Neurology, Warsaw, Poland; 2 1st Department of Cardiology, Medical University of Warsaw, Warsaw, Poland; 3 Department of Experimental and Clinical Pharmacology, Medical University of Warsaw, Warsaw, Poland

## Abstract

**Aim:**

To test if circulating levels of markers of inflammation, endothelial function, and chronic infections, as well as association between these markers and carotid intima media thickness (CIMT), depend on the stage of atherosclerosis expressed as a history of a major vascular event.

**Methods:**

The associations were analyzed separately in 75 healthy controls, 79 patients 3-6 months after the first-ever non-cardioembolic ischemic stroke (IS), and 37 patients 3-6 months after the first-ever myocardial infarction (MI). Data were collected prospectively in 2005. We measured high sensitivity C-reactive protein (hs-CRP), procalcitonin, E-selectin, intercellular adhesion molecule-1 (ICAM-1), serum level of immune complexes (IC), and identified antibodies against Herpes simplex virus type 1 (HSV), Cytomegalovirus, Chlamydia pneumonia, and *Helicobacter pylori*. Correlations with CIMT were determined using Pearson R and verified after adjustment for age, sex, hypertension, diabetes, and statin therapy.

**Results:**

Median ICAM-1 concentration was significantly lower in controls than in post-IS patients (188 μg/L vs 215 μg/L), and significantly lower in post-IS patients than in post-MI patients (215 μg/L vs 260 μg/L). Control patients also had significantly lower IC level (0.03 U/L) and HSV antibody index (6.0) compared to both post-IS (0.6 U/L, 9.6) and post-MI (0.4 U/L, 9.2) patients. CIMT was correlated with age (Pearson R = 0.38, *P* = 0.001) in the control group, immune complexes (R = 0.26, *P* = 0.023) in the post-IS group, and with hs-CRP (R = 0.40, *P* = 0.017) in the post-MI group. These correlations were confirmed using multiple regression analysis.

**Conclusions:**

Our study supports linear correlations between CIMT and IC and hs-CRP levels. However, these associations seem to depend on the type of vascular burden.

Atherosclerosis is a chronic inflammatory and metabolic disease that may manifest as myocardial infarction (MI), ischemic stroke (IS) or other conditions caused by arterial stenosis. These acute events are usually triggered either by progression to a flow-limiting disease or thrombus formation on vulnerable plaque (1,2). Despite major advances in the prevention and treatment, stroke and coronary artery disease remain the leading causes of morbidity worldwide, accounting for approximately one third of all deaths (3).

There are currently several pathological, biochemical, and imaging criteria proposed to identify high risk plaques and high risk patients (4). Atherosclerotic plaques and carotid intima-media thickness (CIMT) are widely accepted as measures of the degree of atherosclerosis. Although highly intercorrelated, they probably reflect different biological aspects and stages of the disease (5). The presence of plaque indicates a higher risk of acute ischemic events (6,7), while CIMT without plaque is a significant marker of atherosclerosis and predicts plaque development (6). Both CIMT and the degree of stenosis may improve the prediction of cerebrovascular events in patients with asymptomatic carotid stenosis (8). This refers especially to the subpopulations with intermediate or high vascular risk (9).

Despite advances in visualization and grading of coronary atherosclerosis, there is still a need to identify additional groups of patients who may benefit from early initiated aggressive vascular prevention (10,11). Published data suggests that systemic factors play an important role in the progression of atherosclerosis and may contribute to plaque instability (2,4). However, the evidence supporting particular serum biomarkers is mixed and inconclusive, which dampened early enthusiasm (2,4). The usefulness of incorporating them into predictive models based on conventional vascular risk factors has not been confirmed (12).

Our aim was to investigate the relationship between CIMT and circulating levels of markers of inflammation, endothelial function, and chronic infections in three different clinical stages of atherosclerosis. We chose the common markers that were already shown to increase in the acute phase of stroke or MI (12,13), but their levels in the chronic phase after vascular event have not been well established. We hypothesized that the levels of at least some of these biomarkers would be lower in control patients compared to patients after IS of non-cardioembolic etiology or after MI. We also identified correlations between CIMT and investigated biomarkers.

## Materials and methods

### Patients

The study was approved by the Committee for Ethics in Human Research at the Institute of Psychiatry and Neurology in Warsaw, Poland and carried out in year 2005 as a part of larger interdisciplinary research project. Patients with a history of IS were recruited from a single stroke center, which provides neurological care for approximately 250 000 inhabitants of a highly urbanized area (Warsaw). The diagnosis of stroke was based on clinical symptoms according to the WHO definition (14) and brain imaging (usually non-contrast computed tomography). The patients were eligible for inclusion if (i) 3 to 6 months had passed after the first-ever ischemic stroke, (ii) they were between 55 and 85 years old, (iii) had a negative history of atrial fibrillation or intracardiac thrombi, (iv) had a negative history of clinically manifest or silent MI, (v) had a negative history of cancer, and (vi) had no signs of an ongoing infection.

Patients with a history of MI were recruited from a single department specialized in hypertension, cardiology, and general medicine, which provides care for approximately 500 000 inhabitants of a highly urbanized area (Warsaw). The patients were eligible for inclusion if (i) 3 to 6 months had passed after the first-ever MI, (ii) they were between 55 and 85 years old, (iii) had a negative history of previous stroke, (iv) had none or mild symptoms of congestive heart failure (class I or II of the New York Heart Association Functional Classification), (v) had a negative history of cancer, and (vi) had no signs of an ongoing infection.

The control group included other patients admitted to both centers and healthy volunteers recruited from staff and their families. The inclusion criteria were (i) age between 55 and 85 years, (ii) negative history of stroke, (iii) negative history of MI or coronary artery disease, (iv) negative history of connective tissue disease, (v) negative history of cancer, and (vi) no signs of an ongoing infection.

Data about vascular risk factors and concomitant medications were collected using a predefined form.

### Biochemical assays

Investigated biomarkers included markers of inflammation (high-sensitivity C-reactive protein [hs-CRP], procalcitonin, [PCT]), markers of endothelial function (intercellular adhesion molecule-1 [ICAM-1], E-selectin), and markers of chronic infection (immune complexes [IC]; the presence of antibodies to Herpes simplex virus type 1 [HSV-1], Cytomegalovirus [CMV], Chlamydia pneumoniae lipopolysaccharide, and *Helicobacter pylori* in IC).

Fasting venous blood samples were collected from the patients who had no evidence of infection, centrifuged, and stored at -80°C. CRP was measured using immunonephelometric method (Dade Behring N Latex High Sensitivity CRPTM mono assay on a Behring Nephelometer 100 analyzer, Marburg, Germany). PCT was assessed using the immunoluminometric assay (LUMItest PCT, BRAHMS Diagnostica, Berlin, Germany).

Endothelial markers were measured with ELISA using commercially available kits (R&D Systems, Minneapolis, MN, USA for ICAM-1, and Amersham Bioscience, Amersham, UK for E-selectin). Absorbance at 450 nm was assessed on a Stat-Fax 2100 microplate reader (Awareness Technology Inc., Palm City, FL, USA).

The IC level was determined by selective precipitation of immune complexes with polyethylene glycol (PEG) followed by an ELISA assay to detect the specific antigens presence in the precipitate, as described previously (13). Briefly, 0.3 mL of the serum sample was added to the 6 mL of 5% PEG in sodium borate buffer, pH 8.4, incubated overnight at 4°C, and centrifuged at 2500 rpm for 20 minutes at 40°C. Pellets were washed twice with 3.5% PEG and dissolved with 0.3 mL distilled water with the addition of 2.7 mL of 0.1 mol/L sodium hydroxide. The blind sample consisted of 0.3 mL distilled water and 2.7 mL of 0.1 mol/L sodium hydroxide. The extinction was measured on a spectrophotometer at 280 nm. The results were expressed as OD_280_ and considered positive if exceeded the geometric mean of OD_280_ (calculated from the log-transformed distribution).

The level of IgG antibodies to *Helicobacter pylori*, Chlamydia pneumoniae lipopolysaccharide, CMV, and HSV-1 in IC were measured using commercial ELISA kits, according to manufacturer’s protocol. Levels of IgG antibodies to H. pylori (Ridascreen ELISA test, R-Biopharm AG, Darmstadt, Germany) were expressed in units per liter (U/L). The levels of IgG antibodies to C. pneumoniae, CMV, and HSV-1 (VIRCELL, Granada, Spain) were expressed as indices relative to the concentrations of antibodies (antibody index = sample optical density/cut off serum mean optical density), as provided by the manufacturer. Serum cholesterol and triglyceride levels were measured using routine commercial laboratory assays.

### Carotid ultrasound

All examinations of CIMT and the degree of carotid stenosis were performed by a single experienced sonographist (BB-H), as described in detail elsewhere (15). Briefly, the participant was supine in a dark room, and the examinations were done with the head held in the midline position or slightly tilted to either side. IMT was defined as the distance between the lumen-intima and the media-adventitia of the common carotid artery (CCA) with a clearly identified double-line pattern. All measurements were done at the far wall on a 1-cm segment of a distal and free-of-plaque part of CCA, using Siemens Acuson 128 XP/10 C scanner with 7 MHz probe (Munich, Germany) (6). Semi-automated edge detection was used to obtain average CIMT across the entire distance at a resolution of 0.1 mm. Each measurement was repeated three times. Because atherosclerosis is usually not symmetrically distributed between the left and right CCA, we used the mean CIMT from both CCAs as a single composite measure of atherosclerosis. CIMT assessment was limited to CCA, as measurements taken in the carotid bifurcation and internal carotid artery are considered less reproducible and more likely to be the source of missing data (16).

### Statistical analysis

**Figure 1:**
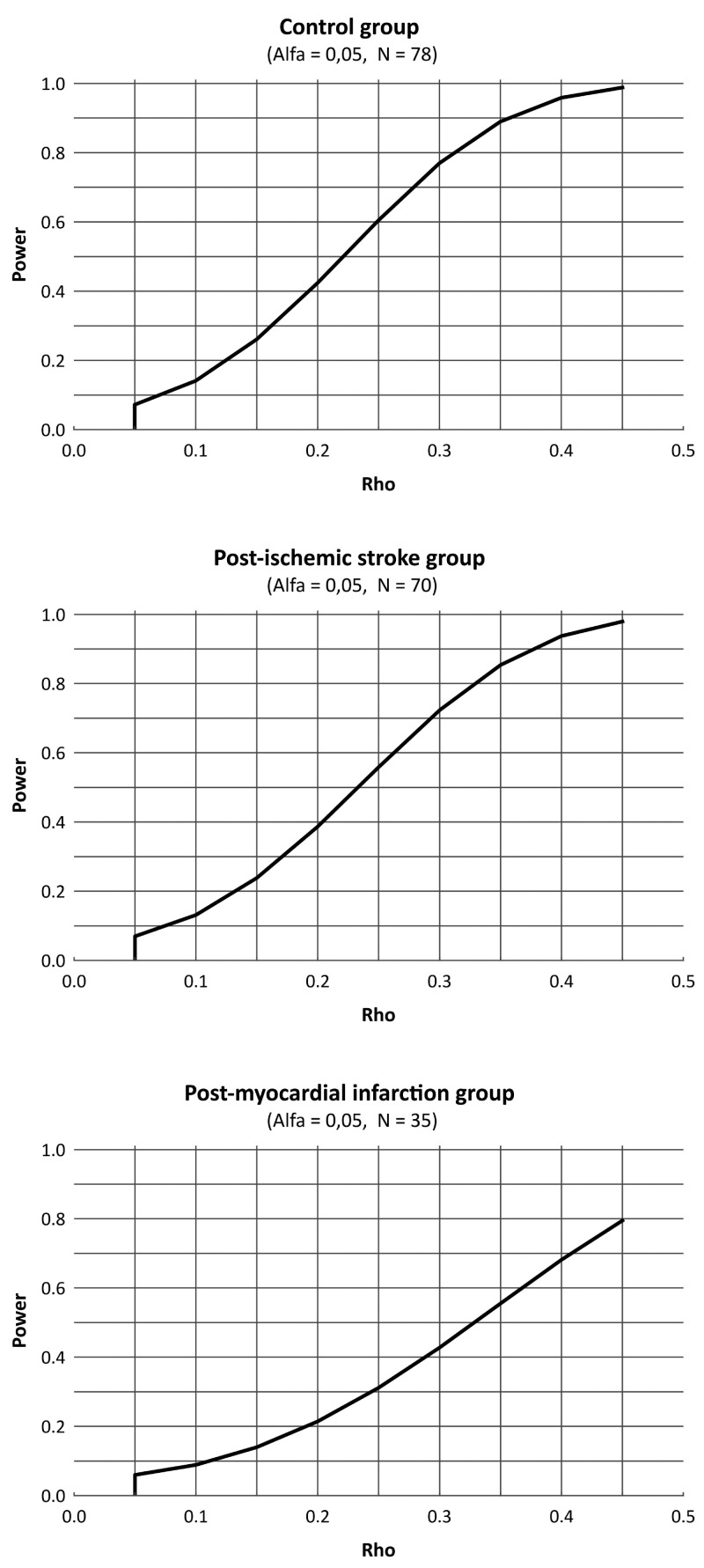
Relation of power to Pearson rho in three groups of participants.

Categorical variables are presented as the number of valid observations with corresponding proportions. Due to non-normal distribution, verified by Shapiro-Wilk test and visual assessment of histograms, continuous variables are presented as a median with the interquartile range (IQR). We applied Box-Cox transformation, which enabled the use of parametric statistics. Comparisons were made using χ^2^ test (with Yates correction if any of the expected values in a 2 × 2 contingency table was <5) and one-way ANOVA. If the overall test for significance across all three groups was positive (*P* < 0.05), *post hoc* pairwise comparisons were done using χ^2^ tests or *t* tests, as appropriate. Correlations between particular biomarkers and CIMT were assessed with Pearson rho (R) coefficient separately in each group of patients. They were subsequently verified in multiple regression models adjusted for age, sex, hypertension, diabetes, and the use of statins. Data collection was not preceded by power calculation but to improve the external validity of obtained results, we additionally calculated power according to rho for the lowest number of observations in each study group (Figure 1). STATISTICA software package ver. 10.0 (Stat Soft Inc., Tulsa, OK, USA) was used and a *P* value of <0.05 was considered significant.

## Results

### Group comparisons

Among 194 participants there were 76 (39.2%) controls without any past ischemic event, 80 (41.2%) post-IS patients, and 38 (19.6%) post-MI patients. All groups were balanced in terms of age, sex, and smoking status (Table 1). Compared to the control group, patients from both post-IS and post-MI group were significantly more often hypertensive and diabetic; more often used antiplatelets, antihypertensives, and statins; and had higher mean CIMT (Table 1). The proportion of patients with >60% internal carotid stenosis was highest in the post-IS group (Table 1). The post-MI group showed the highest prevalence of congestive heart failure and atrial fibrillation, the highest use of antihypertensives and statins, as well as the lowest level of total cholesterol and the lowest alcohol consumption (Table 1).

**Table 1:** Patients’ characteristics in three groups of participants

	Control group		Post-ischemic stroke group		Post-myocardial infarction group		Overall *P*	Pairwise differences*
	n	value	n	value	n	value		
Carotid intima media thickness (mm), median (IQR)	76	0.80 (0.70; 1.00)	80	1.00 (0.90; 1.10)	38	0.90 (0.80; 1.20)	<0.001	cs, cm
**Basic characteristics**								
age, median (IQR)	76	62.5 (58; 72)	80	66 (60; 72.5)	38	67.5 (62; 73)	0.141	
women, No. (%)	76	43 (56.6)	80	33 (41.3)	38	21 (55.3)	0.123	
arterial hypertension, No. (%)	76	36 (47.4)	80	57 (71.3)	38	28 (73.7)	0.002	cs, cm
congestive heart failure, No. (%)	76	3 (4.0)	80	2 (2.5)	38	11 (29.0)	<0.001	cm, sm
atrial fibrillation, No. (%)	76	1 (1.3)	80	0 (0.0)	38	4 (10.5)	0.002	cm, sm
coronary artery disease, No. (%)	76	0 (0.0)	80	18 (22.5)	38	38 (100.0)	<0.001	cs, cm, sm
internal carotid artery stenosis >60%, No. (%)	76	2 (2.6)	80	16 (20.0)	38	2 (5.3)	0.001	cs, sm
diabetes, No. (%)	76	10 (13.2)	80	21 (26.3)	38	13 (34.2)	0.025	cs, cm
current smoking, No. (%)	73	28 (38.4)	77	34 (44.2)	38	13 (34.2)	0.558	
alcohol consumption, No. (%)	67	27 (40.3)	71	38 (53.5)	36	5 (13.9)	<0.001	cm, sm
total cholesterol (mmol/L), median (IQR)	71	5.6 (4.9; 6.6)	76	5.3 (4.6; 6.3)	32	4.9 (4.0; 5.6)	0.002	cm, sm
triglycerides (mmol/L), median (IQR)	61	1.3 (0.9; 1.7)	67	1.2 (0.9; 1.7)	28	1.5 (1.0; 1.8)	0.361	
**Medications**								
antiplatelets, No. (%)	76	15 (19.7)	80	62 (77.5)	38	35 (92.1)	<0.001	cs, cm
antihypertensives, No. (%)	76	32 (42.1)	80	47 (58.8)	38	34 (89.5)	<0.001	cs, cm, sm
statins, No. (%)	76	6 (7.9)	80	40 (50.0)	38	35 (92.1)	<0.001	cs, cm, sm
oral anticoagulants, No. (%)	76	1 (1.3)	80	4 (5.0)	38	4 (10.5)	0.086	

*cs – significant difference between control group and post-ischemic stroke group; cm – significant difference between control group and post-myocardial infarction group; sm – significant difference between post- ischemic stroke and post-myocardial infarction group; IQR – interquartile range.

Median concentration of ICAM-1 was lowest in controls (188 μg/L), significantly higher in post-IS group (215 μg/L), and then significantly higher in post-MI group (260 μg/L). Compared to controls, both post-IS and post-MI groups showed significantly higher levels of IC and HSV-1 antibodies. Levels of other biomarkers were similar across all groups. There was a tendency toward higher levels of E-selectin in the post-IS group and particularly in post-MI group but it did not reach significance (Table 2).

**Table 2:** Serum biomarkers in three groups of participants

	Control group		Post- ischemic stroke group		Post-myocardial infarction group		Overall *P*	Pairwise differences*
	n	median (IQR)	n	median (IQR)	n	median (IQR)		
High sensitivity C-reactive protein (mg/L)	75	2.1 (1.3; 3.5)	79	2.6 (1.5; 5.5)	35	2.8 (1.5; 6.8)	0.156	
Procalcitonin (μg/L)	74	0.00 (0.00; 0.11)	78	0.00 (0.00; 0.10)	37	0.00 (0.00; 0.10)	0.660	
Intercellular adhesion molecule-1 (μg/L)	70	188 (136; 250)	79	215 (160; 295)	35	260 (218; 350)	<0.001	cs, cm, sm
E-selectin (μg/L)	70	8.2 (4.0; 13.0)	79	10.5 (4.5; 20.0)	35	14.5 (6.0; 21.5)	0.131	
Immune complexes (U/L)	75	0.03 (0.02; 0.03)	79	0.06 (0.03; 0.11)	37	0.04 (0.03; 0.09)	<0.001	cs, cm
*Helicobacter* antibodies (U/L)	75	1.0 (0.6; 3.5)	79	2.0 (0.9; 5.3)	35	2.3 (0.6; 5.0)	0.101	
Herpes simplex virus type 1 (antibody index)	75	6.0 (4.8; 9.1)	78	9.6 (7.5; 12.1)	37	9.2 (6.5; 10.8)	<0.001	cs, cm
Cytomegalovirus (antibody index)	73	7.1 (3.4; 12.6)	79	7.1 (3.9; 12.9)	35	8.4 (5.0; 12.5)	0.769	
Chlamydia (antibody index)	72	6.4 (4.3; 11.3)	79	7.6 (3.3; 12.7)	35	8.6 (4.5; 13.4)	0.394	

*cs – significant difference between control group and post-ischemic stroke group; cm – significant difference between control group and post-myocardial infarction group; sm – significant difference between post- ischemic stroke and post-myocardial infarction group; IQR – interquartile range.

### Correlations between CIMT and other factors

In the control group, CIMT moderately correlated with age (Pearson R 0.38, *P* = 0.001), which was confirmed in the multivariate model adjusted for age, sex, hypertension, diabetes, and the use of statins, as well as in the sensitivity analysis (Table 3).

**Table 3:** Correlations between carotid intima media thickness and different biomarkers in three groups of participants

	Control group			Post- ischemic stroke group			Post-myocardial infarction group		
	unadjusted	multivariable*		unadjusted	multivariable*		unadjusted	multivariable*	
	Pearson rho	Beta	*P*	Pearson rho	Beta	*P*	Pearson rho	Beta	*P*
**Basic factors**									
age (years)	0.38 (*P* = 0.001)	0.36	0.003	0.09	0.19	0.098	-0.05	-0.19	0.237
cholesterol (mmol/L)	0.07	0.11	0.372	0.03	-0.01	0.922	-0.04	0.01	0.943
triglycerides (mmol/L)	0.15	0.18	0.166	0.11	0.02	0.886	0.38 (*P* = 0.046)	0.33	0.071
**Biomarkers**									
high sensitivity C-reactive protein (mg/L)	0.07	0.01	0.936	0.04	-0.04	0.725	0.40 (*P* = 0.017)	0.38	0.017
procalcitonin (μg/L)	0.08	0.04	0.721	-0.05	-0.05	0.626	0.00	-0.07	0.706
intercellular adhesion molecule-1 (μg/L)	0.09	0.09	0.439	-0.06	-0.08	0.464	0.02	0.23	0.210
E-selectin (μg/L)	0.02	-0.01	0.946	0.09	0.11	0.362	0.00	-0.05	0.732
immune complexes (U/L)	0.10	0.06	0.576	0.26 (*P* = 0.023)	0.23	0.042	-0.06	0.04	0.815
*Helicobacter* antibodies (U/L)	0.03	-0.03	0.820	-0.05	-0.06	0.571	-0.08	0.00	0.983
Herpes simplex virus type 1(antibody index)	0.09	0.02	0.864	-0.07	-0.03	0.770	-0.11	-0.04	0.782
Cytomegalovirus (antibody index)	0.16	0.05	0.662	-0.05	0.01	0.959	-0.06	-0.05	0.789
Chlamydia (antibody index)	0.13	-0.03	0.793	-0.02	-0.06	0.585	0.06	0.05	0.753

*Multiple regression models include age, sex, arterial hypertension, diabetes, and use of statins. Significant values are marked in gray.

In the post-IS group, CIMT modestly correlated with IC level (Pearson R 0.26, *P* = 0.023), which was confirmed after adjustment for age, sex, arterial hypertension, diabetes and use of statins. However, this association lost significance in the sensitivity analysis (Table 3).

In the post-MI group, CIMT moderately correlated with hs-CRP (Pearson R 0.40, *P* = 0.017) and triglycerides (Pearson R 0.38, *P* = 0.046). The correlation with hs-CRP was confirmed both in the multivariate model and sensitivity analysis. However, the correlation with triglycerides became non-significant after adjustment for confounding variables (Table 3). No other associations between CIMT and investigated biomarkers were detected (Table 3).

## Discussion

We found that CIMT was correlated with the level of immune complexes in chronic post-IS patients and with hs-CRP in post-MI patients, while no significant correlations were found in the control group. Thus, our study adds to the existing pool of data about usefulness of several biomarkers of inflammation, chronic infection, and endothelial function in different groups of patients for approximating the degree of atherosclerosis. Their relevance appears to depend on the type of vascular burden.

Intima media is composed of smooth muscle cells (media layer, 80%) and endothelium (intima, 20%), which cannot be distinguished by ultrasound imaging (5). There are still no accepted standards on how to use of IMT in various research areas (15). However, it is generally agreed that early diffused IMT reflects not only atherosclerosis but also compensatory remodeling caused by hypertensive hypertrophic response of the muscle cells to changes in local shear and tensile stress (5,6). On the other hand, plaque is a localized manifestation of atherosclerosis, which is more likely related to inflammation, oxidation, endothelial dysfunction, and⁄or smooth muscle cell proliferation (5).

Carotid and coronary atherosclerosis have common risk factors, including hypertension, smoking, diabetes, older age, and dyslipidemia (17,18). In our study, age was associated with CIMT only in controls. This may be explained by the fact that only individuals with advanced stage of atherosclerosis may experience a non-cardioembolic IS or MI. There was also an unadjusted correlation between CIMT and triglyceride concentration in post-MI patients, whereas the correlation with cholesterol was found in none of the investigated groups. One may speculate that intensive statin therapy initiated in almost every case after MI significantly reduces LDL cholesterol levels. Therefore, high triglycerides may also partly reflect the extent of dyslipidemia that preceded the acute vascular event. On the other hand, the relationship between dyslipidemia and stroke is not clearly established and the preventive role of statins can most likely be attributed to the modulation of endothelial function and inflammatory processes (18-20). It should also be noted that the pleiotropic effect of statin therapy may involve biomarkers and result in CIMT regression (18,20). However, the time from the ischemic event to enrollment was probably too short to allow for significant changes in CIMT.

The histopathology of carotid atherosclerotic is similar to that of coronary atherosclerosis. However, IS results mainly from plaque rupture or embolization from ulcerated plaques, whereas MI more likely results from erosion and static thrombotic occlusion (21). Therefore, the usefulness of particular biomarkers for predicting plaque instability in those two vascular beds may be different (10). In our study there was no significant difference between CIMT in post-IS and post-MI group. These results are in line with data from a large European cohort (22).

### Markers of inflammation

Low-level artery wall inflammation is involved in all stages of atherogenesis. However, despite extensive studies, it has not been fully established whether serum hs-CRP plays an active role or whether it simply reflects other pathological processes (23,24). We found a moderate positive correlation between CIMT and hs-CRP in patients with a history of MI. This correlation was independent of age, sex, hypertension, diabetes, and statin therapy. However, it was not found in the post-IS group and control group. Previous studies showed that higher hs-CRP levels were associated with higher coronary risk but were less likely to indicate high-risk carotid atherosclerosis (23,25-28). Therefore, our results add to the pool of evidence supporting the potential usefulness of CRP for the detection of vulnerable cardiac patients. It should also be noted that the addition of hs-CRP to the predictive model including conventional risk factors from the Framingham score only mildly improved risk discrimination (24).

We did not observe a correlation between CIMT and PCT, a marker of systemic response to bacterial infection. Previous studies showed that PCT may predict long term cardiovascular mortality (29,30). However, PCT was associated with established vascular risk factors and was not superior to hs-CRP (29,30). It also failed to identify patients with unstable carotid stenosis (31).

### Markers of chronic infection

Previous studies suggest that chronic infections may be involved in atherogenesis (32,33). Several bacterial and viral pathogens have been detected in human plaques or in normal vessel wall. However, the exact mechanisms of action and potential causality are still far from being fully understood (32,33). Our findings support the hypothesis that the chronic inflammatory processes provoked or exaggerated by immune complexes and their accumulation in vessel wall may contribute to the development of atherosclerosis, resulting in an ischemic event, particularly stroke. Although HSV antibody index was higher in both vascular groups compared to controls, none of the evaluated pathogens showed clear relationship with CIMT. We are not able to determine which antigens were responsible for the increased IC level. Nonetheless, our study adds negative data to the pool of mixed-positive evidence for C. pneumoniae and mixed evidence for H. pylori, CMV, or HSV (31-33). Of course, our results do not exclude the potential role of these biomarkers in the acute phase of IS or MI (13).

### Markers of endothelial function

Another factor probably involved in the promotion or exacerbation of atherosclerosis is endothelial dysfunction (34-36). However, despite extensive studies this is not supported by strong evidence (34-36). In our study, ICAM-1 levels were lowest in the control group, moderately elevated in post-IS group, and highest in post-MI group. However, we did not find any correlation between ICAM-1 and CIMT in any of the evaluated groups. As for E-selectin, there were neither intergroup differences nor correlations with CIMT. However, previous studies suggest that sICAM-1 may be an early biomarker indicating disturbance of the endothelium and the presence of advanced plaque in the coronary and carotid arteries (37,38). Some studies also report that its concentration increases especially after or in the course of ischemic episode, which may imply its involvement in carotid plaque destabilization (37). ICAM-1 and E-selectin (but not vascular cell adhesion molecule 1) have also been shown to predict carotid artery atherosclerosis and development of coronary heart disease (39). This effect can be partly attenuated by the use of statins, which in turn increases nitric oxide generation and reduces circulating levels of ICAM-1 and P-selectin in hypercholesterolemic patients (40).

### Strengths and limitations

A restricted number of individuals is a common limitation in studies investigating multiple biomarkers of one pathological condition but it needs to be noted, especially in the post-MI group. The sample size in our study is similar to what has been reported in other biomarker studies (11,31). It was not based on power calculations because the aim of the study was to address several biomarkers without prioritizing any of them. This should be taken into account while interpreting the results, especially in the post-myocardial infarction group. We may not exclude that larger sample size would yield significant correlations between CIMT and other biomarkers. However, to make the analysis more robust we applied Box-Cox transformation, which enabled the use of parametric statistics. Our intention was to carry out an exploratory analysis that would identify associations of potential clinical importance. Judging from the point estimates, even if the association was proven significant after doubling or tripling the number of participants, its strength would be low and without any clinical implications. It needs to be noted that the control group consisted of both healthy volunteers and non-stroke or non-coronary artery disease patients, which was intended to better reflect general population but may have also been a source of bias, since it does not ensure full representativeness of the control group for the general non-stroke and non-MI population. Blood samples were taken only once, which may have introduced bias caused by intraindividual variation. Detailed lipid profile was not assessed in all patients and therefore the levels of LDL and HDL cholesterol were not reported. All patients had already entered the chronic stage of disease, meaning that many of them were using statins at least for several months, which may have affected the levels of inflammatory biomarkers. However, the levels measured early after IS and MI do not reflect their everyday levels and clearly decrease over time, which makes them less reliable for long-term risk stratification (13,41). All patients were Caucasians recruited from one geographically homogenous area, which excludes potential ethnical bias (42). They were also subjected to identical diagnostic procedures carried out by the same group of investigators, which made direct comparisons fully justified.

### Conclusions

Considering differences between pathologies leading to symptomatic coronary or carotid ischemic events, it may be difficult to identify a single universal blood marker of atherosclerosis that would significantly improve present clinical predictive models. Nonetheless, there is still need for further well designed and large scale studies of novel biomarkers.
